# A neuronal theta band signature of error monitoring during integration of facial expression cues

**DOI:** 10.7717/peerj.12627

**Published:** 2022-02-17

**Authors:** Camila Dias, Diana Costa, Teresa Sousa, João Castelhano, Verónica Figueiredo, Andreia C. Pereira, Miguel Castelo-Branco

**Affiliations:** 1 CIBIT - Coimbra Institute for Biomedical Imaging and Translational Research, University of Coimbra, Coimbra, Portugal; 2 ICNAS - Institute for Nuclear Sciences Applied to Health, University of Coimbra, Coimbra, Portugal; 3 FMUC - Faculty of Medicine, University of Coimbra, Coimbra, Portugal

**Keywords:** Error monitoring, Theta oscillations, EEG, Eye tracking, Facial Cue integration

## Abstract

Error monitoring is the metacognitive process by which we are able to detect and signal our errors once a response has been made. Monitoring when the outcome of our actions deviates from the intended goal is crucial for behavior, learning, and the development of higher-order social skills. Here, we explored the neuronal substrates of error monitoring during the integration of facial expression cues using electroencephalography (EEG). Our goal was to investigate the signatures of error monitoring before and after a response execution dependent on the integration of facial cues. We followed the hypothesis of midfrontal theta as a robust neuronal marker of error monitoring since it has been consistently described as a mechanism to signal the need for cognitive control. Also, we hypothesized that EEG frequency-domain components might bring advantage to study error monitoring in complex scenarios as it carries information from locked and non-phase-locked signals. A challenging go/no-go saccadic paradigm was applied to elicit errors: integration of facial emotional signals and gaze direction was required to solve it. EEG data were acquired from twenty healthy participants and analyzed at the level of theta band activity during response preparation and execution. Although theta modulation has been consistently demonstrated during error monitoring, it is still unclear how early it starts to occur. We found theta power differences at midfrontal channels between correct and error trials. Theta was higher immediately after erroneous responses. Moreover, before response initiation we observed the opposite: lower theta preceding errors. These results suggest theta band activity not only as an index of error monitoring, which is needed to enhance cognitive control, but also as a requisite for success. This study adds to previous evidence for the role of theta band in error monitoring processes by revealing error-related patterns even before response execution in complex tasks, and using a paradigm requiring the integration of facial expression cues.

## Introduction

Error monitoring is an executive function skill that plays a crucial role in adaptive human behavior since it signals the need for performance improvement (Kim et al., 2018). It can be operationally defined as a cognitive process that reflects the ability to monitor one’s actions. Upon execution, an internally generated monitoring system compares a representation of the expected response with a representation of the actual one. It allows our actions to be shaped by their outcomes both in the short term, for example, by responding more cautiously to avoid further errors, and in the long term, through gradual learning of appropriate stimulus–response contingencies (Ullsperger, Danielmeier & Jocham, 2014). In fact, impairments in error monitoring processes are implicated in several brain disorders, particularly in those where impulsive behaviors are common, such as obsessive-compulsive disorder (Riesel, 2019), schizophrenia (Bates et al., 2009), autism spectrum disorder (ASD) (Santesso et al., 2011), anxiety (Meyer, 2016), depression, and substance abuse (Olvet & Hajcak, 2008).

Here, we seek to determine the neuronal signatures of such error monitoring processes during the integration of facial expression cues and using electroencephalography (EEG). The recognition and integration of different facial cues are inherently present in our daily interactions. Therefore, a testing paradigm based on such cues might allow going a step further on the approximation of performance monitoring studies to real-life complex scenarios, which assumes increased importance in disease. Moreover, we aimed to study it at the level of theta band oscillations. Brain oscillations at different frequencies provide temporal and spatial codes that may inform us about the dynamics of functional networks of complex integrative functions and are not limited to phase-locked signals. Midfrontal theta oscillations (4–8 Hz), in particular, are believed to reflect error monitoring mechanisms once their power has been consistently found to be increased during erroneous responses (peaking at the FCz location) (Cavanagh, Zambrano-Vazquez & Allen, 2012; Chavarriaga, Sobolewski & Millán, 2014; Cohen, 2011; Pavone et al., 2016; Völker et al., 2018; Zhang et al., 2015).

Midfrontal theta activity has been linked to the processing of conflict (Cohen & Cavanagh, 2011) and unexpected feedback (Van Noordt et al., 2017), and to the experiencing of undesirable action outcomes (Cohen, Elger & Ranganath, 2007). This activity seems to signal a need for increased cognitive control and attention allocation (Van Noordt et al., 2017), essential for the adaptation of behavior (Cohen, 2011). Its neuronal source is estimated to be in the anterior cingulate cortex (ACC) (Iannaccone et al., 2015; Pavone et al., 2016), which plays a central role in decision making and conflict/error monitoring, namely within social contexts (Santesso et al., 2011). There is evidence that theta oscillations reflect a mechanism of interaction by which the ACC signals the need for adaptive changes in cognitive control, which are implemented by the lateral prefrontal cortex (Cohen & Cavanagh, 2011). As such, midfrontal theta has been used as a cortical signature of error monitoring as inferred from frequency analysis of cortical oscillations.

Midfrontal theta has also been linked to the error-related negativity (ERN), a negative event-related potential that arises 50–100 ms after erroneous responses at midfrontal electrode positions (Bhattacharyya et al., 2017; Chavarriaga, Sobolewski & Millán, 2014). It has been suggested that this negativity is elicited whenever an outcome is worse than expected (Moser et al., 2013; Plewan et al., 2016). Nevertheless, given that the response monitoring system is also manifested on correct trials by the correct-related negativity (CRN)—similar to the ERN regarding its timing and topographic distribution but smaller in amplitude (Kim et al., 2018; Vlamings et al., 2008)—there is a growing consensus that the ERN reflects a continuous process of performance monitoring that is augmented after erroneous responses (Vlamings et al., 2008). ERN is followed by a medial parietal positive peak, the Error Positivity (Pe), centered at the Pz electrode, which arises 200–400 ms after response onset. Pe seems to reflect the conscious recognition of errors (Pavone et al., 2016; Völker et al., 2018).

Although there is large evidence concerning the midfrontal theta recruitment during performance monitoring, it is still not clear how early its modulation starts to occur. The increase in theta power following errors, as well as the ERN, might reflect abnormal processing that possibly initiates before an erroneous response (Schroder et al., 2017). In fact, most errors are preceded by a decline in attention, which has been suggested by functional magnetic resonance imaging (fMRI) studies. These have shown that errors are preceded by increased default-mode network (DMN) activity, which is associated with idle states (Ray Li et al., 2007). However, the role of theta oscillations during response preparation remains uncertain. There is some evidence of a decrease in midfrontal theta activity before erroneous responses—this finding has been demonstrated when errors were induced by boredom and mind-wandering (Atchley, Klee & Oken, 2017), during a Flanker task (Cavanagh, Cohen & Allen, 2009), and a saccade task (Van Noordt et al., 2017). However, additional studies are required to clarify this question. As such, the main goal of our study was to investigate whether midfrontal theta modulation occurs only due to error commission or also when preceding an erroneous action.

Furthermore, the great majority of studies concerning the neuronal signatures of the error monitoring system are based on simple tasks with rigidly controlled conditions and time-locked events, which lead to high signal-to-noise ratios and avoid typical confounds of realistic scenarios (Chavarriaga, Sobolewski & Millán, 2014). Spüler & Niethammer (2015) revealed the challenge of recognizing errors in asynchronous events, but demonstrated that the analysis of oscillatory activity allows for this recognition. Our study was based on a go/no-go saccade task with large variability of response timing, and we aimed to understand if the theta band activity allows the discrimination between correct and erroneous responses in this realistic context.

Accordingly, we hypothesized a theta oscillations role not only as alarm signs for the need for further performance improvement but also as a moderator for success. Moreover, we followed the hypothesis that theta activity is suited to study error monitoring in complex scenarios as it contains information from locked and non-phase-locked signals.

## Materials & Methods

### Participants

Twenty healthy participants (nine female, mean age 26.80 ± 4.51 years) were recruited for this study. Our sample size was estimated using G*Power 3.1 (Faul et al., 2007). We have run a sample size estimation for the difference between two dependent means (paired *t*-test) with an effect size of 0.8 and an error probability of 0.05, which resulted in a sample size of 19. Without the normality assumption of the distribution of the differences of the means, we would need 20 subjects for a non-parametric test.

All participants except one were right-handed, and all had normal or corrected-to-normal visual acuity. Every participant provided written informed consent in accordance with the Declaration of Helsinki prior to participation, and the study followed the safety guidelines for research on humans. The work was approved by the Ethics Committee of the Faculty of Medicine of the University of Coimbra approval number CE-001/2021.

### Task

**Figure 1:**
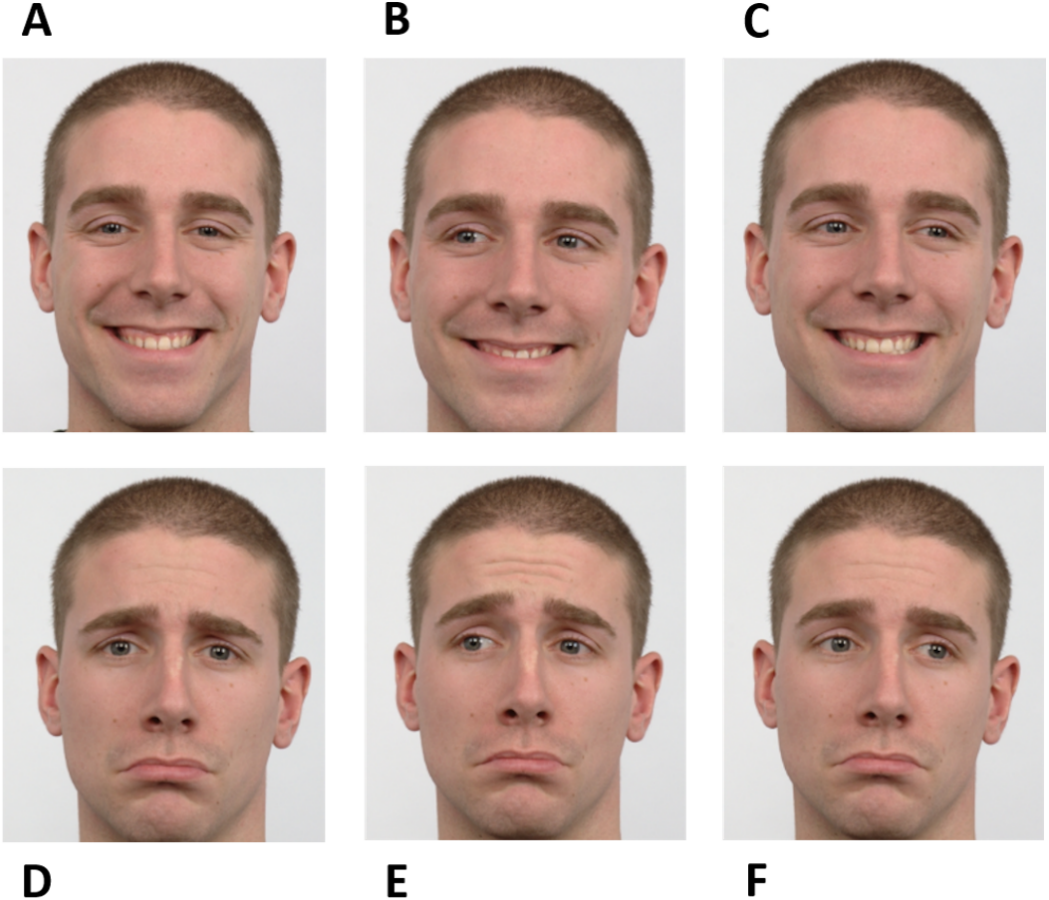
Facial instructions used in the go/no-go saccadic task. The experiment was based on six facial instructions as illustrated: happy no-go (A), left pro-saccade (B), right pro-saccade (C), sad no-go (D), left anti-saccade (E), and right anti-saccade (F). Image source: Langner et al. (2010).

The experiment was based on a go/no-go saccadic task. Facial cues were used as instructions. Instructions were planned to raise the participants’ simultaneous attention to the eyes and mouth of the face presented in the stimuli to achieve high performance. During “go” trials, participants should perform a pro-saccade or an anti-saccade. A happy averted face was the directive to make a pro-saccade, *i.e.,* to look in the same direction of the face shown, while a sad averted face informed the participants to execute an anti-saccade, *i.e.,* to look in the opposite direction of the face shown. The “no-go” trials were signaled by a face (happy or sad) looking straight ahead. Therefore, there were six different instructions according to different combinations of facial expressions and gaze directions - happy no-go, sad no-go, right pro-saccade, left pro-saccade, right anti-saccade, and left anti-saccade (Fig. 1).

**Figure 2:**
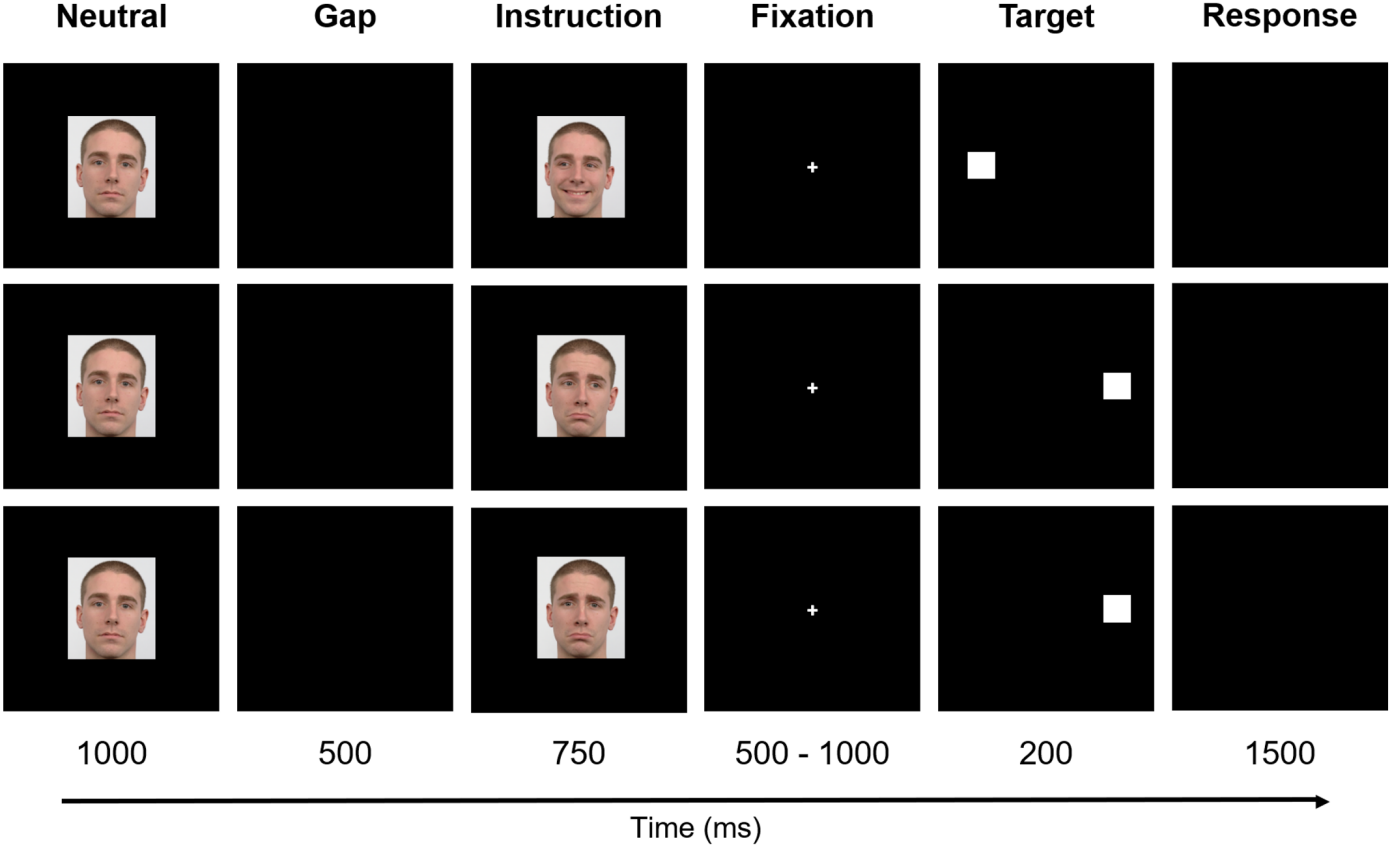
Experimental design. The paradigm included six stages: Neutral, Gap, Instruction, Fixation, Target, and Response. Firstly, a Neutral face was exhibited and followed by a Gap period. Afterward, the Instruction was given. The subsequent stage—Fixation—consisted of the appearance of a central cross to which the subjects were instructed to look. The cross was, then, replaced by a square, the Target, that appeared either on the right or left part of the screen. Finally, a black empty window denotes the period during which the participants were advised to perform the Response. For a better visualization, the image is not to scale. Facial images source: Langner et al. (2010).

The paradigm included six stages - Neutral, Gap, Instruction, Fixation, Target, and Response (Fig. 2). Firstly, the preparatory cue - a Neutral face - was exhibited for one second and followed by a Gap period that lasted 500 ms (black background). Afterward, during a period of 750 ms, the Instruction was given, *i.e.,* one of the six facial expressions illustrated in Fig. 1 was shown (randomly selected). The subsequent stage - Fixation - consisted of the appearance of a central cross for a variable time interval between 500 ms and 1,000 ms (randomly chosen between {500, 600, 700, 800, 900, 1,000} ms to prevent anticipation), to which the subjects were instructed to look during this period. The cross was then replaced by a square, the Target, which appeared on either the right or left part of the screen, coherent with the gaze direction (except when the face was looking forward - in these cases, the target position was randomly placed either on the right or left), during 200 ms. Lastly, a black empty window characterizes the period during which the participants were advised to perform the Response (saccade or no-go), which lasted 1,500 ms.

The stimuli were presented against a black background on a 17-inch monitor (1,280 × 1,024 pixel resolution) with a refresh rate of 60 Hz. The computer screen was placed at a distance of 60 cm in front of the participants. The facial expression images were presented with similar parameters: 4.55°eight and 4.90°width (visual angle), and mean luminance of 7.67 × 10^1^ cd/m^2^ (with screen luminance ranging from 2.44 × 10^−1^ cd/m^2^ to 1.76 × 10^2^ cd/m^2^), measured with Apacer AL100/AL110 Spectroradiometer (version 3.0). The fixation cross and target had lengths of 0.72°and 1.52°of visual angle, respectively. The facial expression images and fixation cross were displayed in the center of the screen, and the target was exhibited with a horizontal distance of 8.30°from the center, either to the right or to the left. The stimuli were programmed in Presentation software (version 12.0, Neurobehavioral Systems Inc.) and the facial expression images of a white young adult male were obtained from the Radboud Faces Database (Langner et al., 2010). All faces had a recognisability higher or equal to 92%.

The task comprised four runs of 84 trials each (28 pro-saccade, 28 anti-saccade, and 28 no-go trials). Happy and sad, right and left trials were counterbalanced. For the no-go conditions, there were 14 sad trials and 14 happy trials. For the pro-saccade and anti-saccade conditions, there were 14 trials in which the gaze was directed to the right and 14 trials in which the gaze was directed to the left. Each run lasted approximately 7 min and began with the calibration of the eye-tracker (ET).

### EEG and ET recording

The experiments were conducted in a quiet room, with sound and light isolation. Skin preparation was performed with the application of abrasive gel and alcohol at 96% to keep electrode impedances below 20 kΩ. EEG and electrooculogram (EOG) were recorded from 64 channels (QuickCap, NeuroScan, USA) with an extended international 10–20 system montage at a sampling rate of 1,000 Hz. Two EOG electrodes were placed at the outer canthi of both eyes (horizontal EOG), and the other two were placed above and below the left eye in a bipolar montage. A central channel close to Cz was used as an online EEG reference during recordings. EEG signals were amplified using a SynAmps 2 system amplifier (Compumedics NeuroScan, Houston, TX, USA) and recorded using Curry Neuroimage 7.08 (NeuroScan).

The ET data were acquired simultaneously with the EEG. Each run started with a 9-point calibration of the ET. The ET data were recorded at 120 Hz in a tower-mounted high accuracy (0.25° - 0.5°) monocular ET (iView X™ Hi-Speed, SMI - SensoMotoric Instruments, Teltow, Germany).

EEG and ET recorded data are available in Zenodo repository (https://doi.org/10.5281/zenodo.5608640).

### Saccade detection

Saccadic movement detection was based on the horizontal coordinate of the point at which the participant was looking. The minimum distance from the center, as well as the minimum horizontal eye movement amplitude to account for the presence of a saccade, was 4.04° of visual angle. In addition, a minimum duration of 15 ms was set to identify a saccade; otherwise, it was considered as a micro-saccade. According to the literature, micro-saccades are characterized by a maximum amplitude of 2° (Martinez-Conde et al, 2009). The defined threshold was set higher due to the high sensitivity of the ET to small head movements, which was leading to false detection of saccades. It was also necessary to consider the fact that both vertical and horizontal coordinates change during a blink. Thus, a saccade was only considered if the vertical amplitude was lower than 80% of the horizontal amplitude. The saccade detection algorithm is schematized in supplemental Fig. S1. It was developed in a custom-made MATLAB script (version R2018b, MathWorks) using the ET file originated during data acquisition containing the times and the coordinates (vertical and horizontal) of the point at which the user was looking.

To validate the algorithm performance, we visually analyzed 115 trials from all participants (3–6 trials per participant randomly selected) by examining the ET file. In each trial, we inspected the presence or absence of a saccade and compared the result to the automatic classification, obtaining a detection accuracy of 97.39%.

### Data analysis

#### Removed/missing data

Data from one participant and two runs (out of four) of another were excluded due to problems in synchronization between EEG and ET data. Moreover, one participant completed only two runs due to ET acquisition problems. Finally, one participant failed all trials from a specific instruction, revealing a misunderstanding of such instruction and, therefore, those trials were not included in the analysis (one-third of that participant’s data).

#### Behavioral data analysis

Each response was defined as correct if the first saccade was executed according to the given instruction, and as erroneous in the opposite case. Examples of erroneous responses were pro-saccades instead of anti-saccades or anti-saccades instead of no-go. The trials without any saccade—correct no-go trials or erroneous pro-saccades/anti-saccades trials in which no saccade was executed—were not considered, as the epochs were centered at the moment of saccade execution.

The relative number of errors (*i.e.,* the ratio between the number of errors and the number of trials), as well as the relative number of pro-saccade errors (*i.e.,* the ratio between the number of pro-saccade errors and the number of errors), anti-saccade errors (*i.e.,* the ratio between the number of anti-saccade errors and the number of errors), and no-go errors (*i.e.,* the ratio between the number of no-go errors and the number of errors), were computed per participant.

Mean response timing (defined as the time interval between the onset of the stage “Response” and saccade execution), mean saccadic amplitude, and mean saccade duration were calculated per participant and compared for correct and error trials.

#### EEG data analysis

EEG data were downsampled to 500 Hz and filtered between 0.05 and 45 Hz since high-pass cutoff frequencies lower or equal to 0.1 Hz produce little artifactual effects (Tanner, Morgan-Short & Luck, 2015). We used a zero-phase non-causal finite impulse response filter. Noisy channels, which were defined based on high amplitudes and frequencies (supported by visual inspection), were removed.

There were only a few noisy channels per participant (maximum five) and none of them included the channel of interest (FCz). Subsequently, the electrodes were re-referenced to the average of all EEG (excluding EOG) channels. Since the task demanded the execution of saccades, there were artifacts in the EEG data due to these ocular movements. To minimize its influence, Independent Component Analysis (ICA)—a commonly used method to reduce saccade, blink, and other artifacts (Castelhano et al., 2014; Dimigen, 2019; Keren, Yuval-Greenberg & Deouell, 2010; Sousa et al., 2017)—was used. The recorded EOG supported the search for blink and saccade artifacts. To remove the non-neuronal sources, independent components were inspected following Chaumon, Bishop & Busch (2015). Afterward, the missing channels (previously removed noisy channels) were interpolated (spherical interpolation) to obtain the same number of channels for all participants.

The data were segmented into epochs of 1,000 ms in length locked to the beginning of the saccade and starting 500 ms before the execution of the saccade. Epochs were visually inspected, and noisy trials (0.77 ± 2.01% of the data) were removed, leaving 202.11 ± 36.29 epochs (correct and error trials) per participant for further analysis. These were corrected for a baseline. For event-related potential analysis, the baseline was defined as [−250, 0] ms. However, since theta activity was examined both before and after saccade execution, the baseline for theta power analysis was the mean activity of the second half of the Gap stage (the first half was not included due to possible confounds related to the neutral face presented on the previous stage).

Although the main research question was related to frequency analysis, we also investigated the EEG signal in the time domain. We analyzed the event-related potentials in FCz and Pz on both correct and error trials because these are the electrodes where the ERN and Pe are more evident, respectively (Bhattacharyya et al., 2017; Chavarriaga, Sobolewski & Millán, 2014; Pavone et al., 2016; Völker et al., 2018). The mean voltage of FCz around the peak position of ERN, *i.e.,* within a time window of 70–160 ms, and the mean voltage of Pz around the peak position of Pe, *i.e.,* within a time window of 200–500 ms, were measured and statistically compared between the erroneous and correct responses.

The mean theta power at FCz—the channel where the theta oscillations that mediate error monitoring are more commonly reported (Cavanagh, Zambrano-Vazquez & Allen, 2012; Cohen, 2011; Pavone et al., 2016)—was computed between 4 and 8 Hz and compared between correct and error trials. The mean power spectral density (PSD) was calculated for all epochs. Analyses were performed considering two specific moments - immediately before and after the saccade onset, both comprising 500 ms. Although we did not focus our analysis on the other acquired channels, these served to support our study (in particular to derive topographic maps).

EEG data analysis was performed using EEGLAB toolbox functions (version 2) in a homemade MATLAB script (version R2018b, MathWorks).

### Statistical analysis

The relative number of pro-saccade, anti-saccade, and no-go errors was statistically compared employing a repeated measures ANOVA test (the normality of the datasets’ distribution was tested with a Shapiro-Wilk test). Given that one participant was excluded and another failed all no-go trials (details in section *Participants*), this subject was not accounted for in this test (*N* = 18). Secondly, response timing, saccade amplitude, and saccade timing duration were compared between correct and erroneous responses (*N* = 19) employing Wilcoxon tests (due to the abnormality of the datasets’ distribution, which was tested with a Shapiro–Wilk test).

Neuronal responses to correct and erroneous actions were compared regarding the mean event-related potentials amplitude and theta power (*N* = 19) employing Wilcoxon tests. Moreover, no-go, pro-saccade, and anti-saccade errors were compared in terms of theta power using a Friedman test, due to the datasets’ small size (*N* = 8), since few participants performed all types of errors.

As the number of correct trials was almost 20 times higher than error trials, we applied per participant a permutation-like test approach, in which 20 subsamples were randomly extracted from the dataset of correct trials with the size of the error trials dataset. Given that, in opposition to the pro-saccadic actions, the anti-saccadic and no-go ones demand inhibitory control mechanisms, the pro-saccadic proportion of trials was kept equal between the correct and error datasets in the analysis. Then, 20 tests (each containing data from all participants) were performed - the dataset of error trials was statistically compared to 20 subsamples of correct trials. A minimum confidence level of 95% for at least 80% of the tests was considered. This means that, to consider a sufficiently powered difference, 16 of 20 tests needed to be characterized by a *p*-value lower than 0.05. The effect size was also computed (partial eta-squared *η*^2^ was assessed for repeated measures ANOVA, Cohen’s *d* was measured for t-tests, and $r= \frac{Z}{\sqrt{N}} $ for Wilcoxon tests).

Statistical analyses were performed using IBM SPSS Statistics 25.

## Results

### Behavioral results

We found errors in 5.21 ± 4.81% of the trials (233 errors). Regarding the relative frequency of erroneous responses, no differences were found between no-go, pro-saccade, and anti-saccade errors (*N* = 18, repeated-measures ANOVA, *F* (2,34) = 1.412, *p* = 0.26, *η*^2^ = 0.08).

The responses were performed, on average, 495.69 ± 606.95 ms before the beginning of the Response stage, which corresponds to the Fixation stage. No significant difference was observed between the moment when correct and erroneous responses were given (*N* = 19, 20 Wilcoxon replication tests to estimate power and balance correct and error trials, mean *Z* =  − 0.88 ± 0.31; none were significant, minimum *p* = 0.06, *r* =  − 0.20 ± 0.07). To verify the absence of differences, we run a Bayes factor analysis. The average Bayes factor (BF) for the 20 datasets was 4.37 ± 0.80, which provides moderate evidence for the alternative hypothesis (H_1_) according to Stefan et al. (2019).

The saccades performed during error trials were smaller than those made during correct trials. The difference found between correct and error trials in terms of saccade duration was significant (*N* = 19, 20 Wilcoxon replication tests to estimate power and balance correct and error trials, mean *Z* =  − 2.34 ± 0.29; 85% were significant with minimum *p* = 0.001 and maximum *p* = 0.04, *r* =  − 0.54 ± 0.07): 70.58 ± 20.91 ms and 56.83 ± 15.60 ms for correct and erroneous responses, respectively.

However, the difference regarding saccade amplitude between correct and error trials was not statistically significant (*N* = 19, 20 Wilcoxon replication tests to estimate power and balance correct and error trials, mean *Z* =  − 2.03 ± 0.31; only 65% were significant with minimum *p* = 0.01 and maximum *p* = 0.04, *r* =  − 0.47 ± 0.07). The average BF was 1.22 ± 0.79, which provides anecdotal evidence for H_1_ (Stefan et al., 2019).

These results are summarized in Table 1. Moreover, the complete statistical results of the 20 tests are displayed in supplemental Table S1.

**Table 1:** Summary of behavioral results. For each metric (response timing, saccade amplitude and saccade duration), the mean and standard deviation are presented for correct and erroneous trials, as well as the significance of the difference between correct and erroneous responses. Given that we perfomed 20 tests for each metric to balance correct and erroneous trials, a minimum confidence level of 95% for at least 80% of the tests was needed to consider a difference as statistically significant (at least 16 of 20 tests with *p* ≤ 0.05). To verify the absence of differences, we run Bayes factor analyses, and the average BF are also shown.

Metric	Correct trials	Error trials	Significance	BF
**Response timing**	−497.02 ± 627.74 ms	−448.75 ± 571.69 ms	–	4.37 ± 0.80
**Saccade amplitude**	10.50 ± 2.59°	9.53 ± 2.41°	–	1.22 ± 0.79
**Saccade duration**	70.58 ± 20.91 ms	56.83 ± 15.60 ms	*	–

### Neurophysiological results

### Error-related potentials

Although our main research question is related to frequency analysis, we analyzed our data in the time domain as well. We wanted to verify if the error-related potentials described in the literature (ERN and Pe) were distinguished during errors. We did not find statistically significant differences between correct and erroneous trials regarding either the mean amplitude of FCz around the peak position of ERN (*N* = 19, 20 Wilcoxon replication tests to estimate power and balance correct and error trials, mean *Z* =  − 1.14 ± 0.44; none were significant, minimum *p* = 0.05, *r* =  − 0.26 ± 0.10) or the mean amplitude of Pz around the peak position of Pe (*N* = 19, 20 Wilcoxon replication tests to estimate power and balance correct and error trials, mean *Z* =  − 2.13 ± 0.39; only 65% were significant with minimum *p* = 0.01 and maximum *p* = 0.05, *r* =  − 0.49 ± 0.09). Furthermore, when comparing correct and erroneous trials, we did not differentiate event-related potential peaks. However, the average BF for the ERN was 3.44 ± 1.58 and for the Pe was 1.39 ± 1.01, which provides moderate and anecdotal evidence for H_1_, respectively (Stefan et al., 2019). Supplemental Fig. S2 and supplemental Fig. S3 show the average amplitude for correct and error trials at FCz and Pz, respectively, across all participants.

### Theta band activity is reduced when preceding erroneous responses and increased afterwards

**Figure 3:**
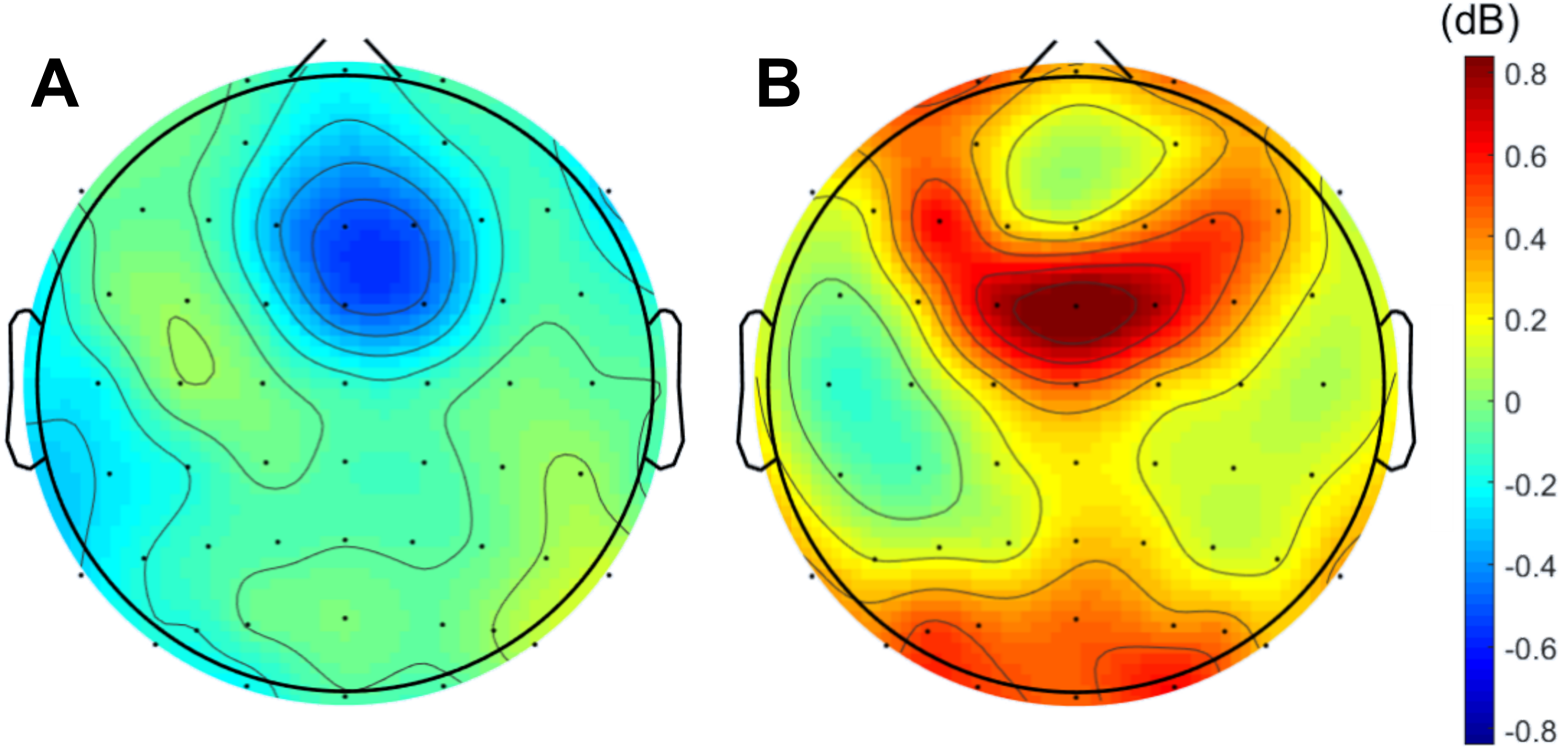
Difference between errors and correct trials regarding theta power before and after participants’ response. Difference (A) before ([-500,0] ms) and (B) after ([0,500] ms) the onset of participants’ response. In both cases, the differences between the neuronal responses recorded at FCz were statistically significant.

**Figure 4:**
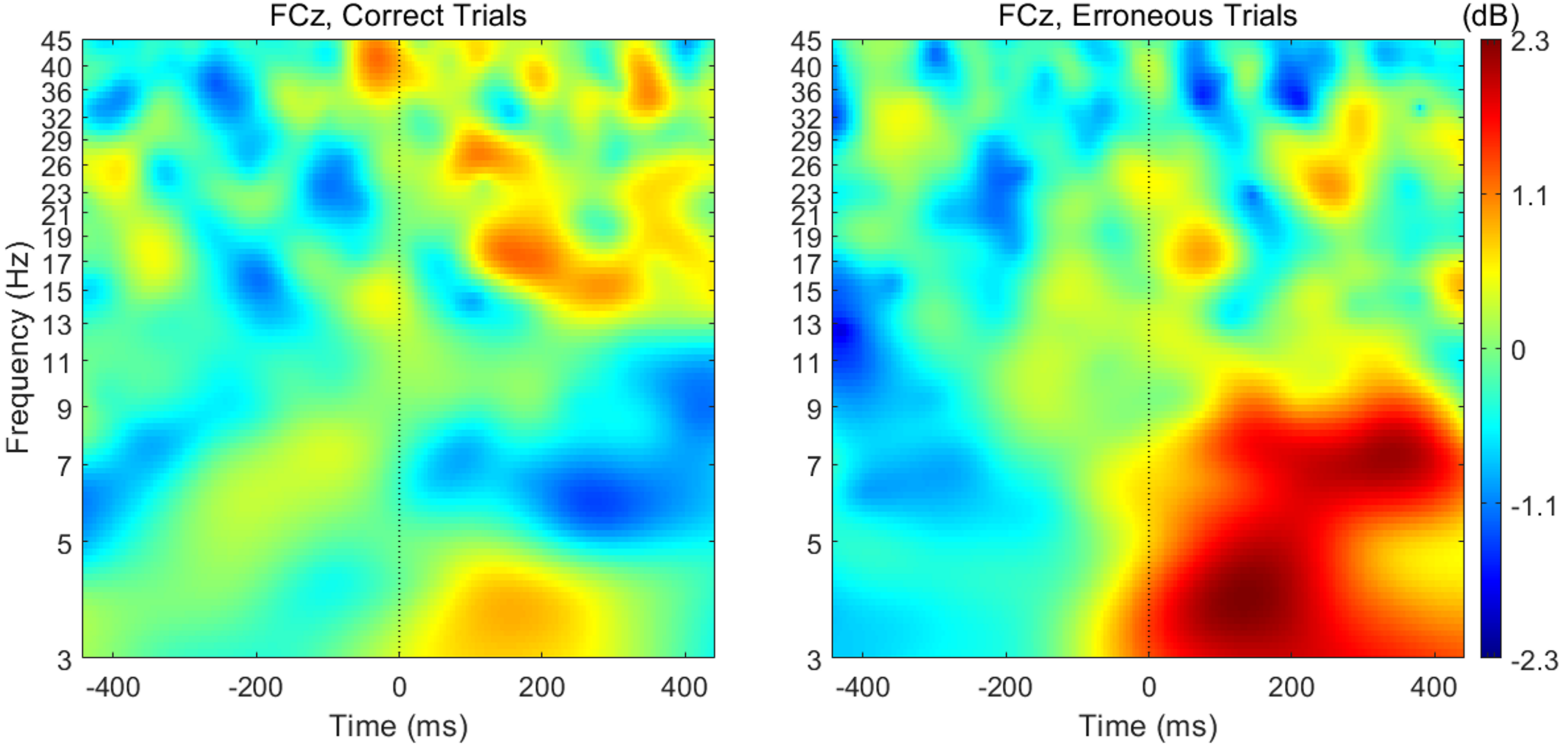
Time-frequency charts for the electrode FCz in correct and error trials.

When analyzing the interval previous to the participants’ responses, we found that theta band power was higher before correct than erroneous responses, in contrast to what happened immediately after errors. Figure 3 illustrates the topographic distribution of the difference between error and correct trials (error minus correct) concerning theta band power before ([−500, 0] ms) and after ([0, 500] ms) the beginning of the participants’ response. An example considering one of the 20 datasets of correct responses (randomly generated from the entire pool of correct responses) is presented. In addition, Fig. 4 shows the time-frequency chart for the electrode FCz during correct (regarding one of the datasets of correct responses) and error trials.

The average theta power at FCz was 1.58 ± 0.85 dB and 1.26 ± 0.60 dB when preceding a correct and an erroneous response, respectively. On the contrary, after the response onset, we found that, on average, theta power was higher during the erroneous (2.31 ± 1.12 dB) than during the correct responses (1.35 ± 0.62 dB). Both comparisons, before (*N* = 19, 20 Wilcoxon replication tests to estimate power and balance correct and error trials, mean *Z* =  − 2.30 ± 0.39; 80% were significant with minimum *p* = 0.004 and maximum *p* = 0.05, *r* =  − 0.53 ± 0.09) and after the onset of the response (*N* = 19, 20 Wilcoxon replication tests to estimate power and balance correct and error trials, mean *Z* =  − 2.11  ± 0.52; 90% were significant with minimum *p* = 0.01 and maximum *p* = 0.05, *r* =  − 0.48 ±0.12), revealed the existence of significant differences between the theta power associated to correct and error trials. The complete statistical results of the 20 replication tests are shown in supplemental Table S2.

When comparing no-go, pro-saccade, and anti-saccade errors separately, we found no significant differences concerning mean theta power at FCz, either before (*N* = 8, Friedman test, *χ*^2^(2) = 0.75, *p* = 0.69) or after (*N* = 8, Friedman test, *χ*^2^(2) = 3.00, *p* = 0.22) the execution of saccades.

## Discussion

In this study, we explored the role of midfrontal theta activity in error monitoring, in particular when facial expression cues need to be integrated. A go/no-go paradigm was applied to study EEG patterns during correct and erroneous responses. We hypothesized that error monitoring processes would be signaled at the level of midfrontal theta power before and after response execution. Accordingly, we found theta power differences at midfrontal channels between correct and erroneous actions before and after response onset.

Regarding the behavioral analysis, we did not find significant differences between correct and erroneous responses, except for the duration of the saccades. The erroneous saccades were significantly shorter than the correct ones, which might be related to the perception of error and the attempt for correction, resulting in unfinished/shorter saccades during error trials. Moreover, the Bayes factor analyses, performed to verify the absence of differences between correct and erroneous response timing, suggested faster responses when they were correctly performed (possibly due to the higher participants’ focus on such cases).

We did not find significant differences between correct and erroneous actions concerning the time domain analysis of the neurophysiological data as well. ERN and Pe, the error-related potentials described in the literature, were not observed during errors. This result was expected for two reasons: a relatively low error rate, and the high variability of saccade timing and duration, which constrained the probability of detecting event-related potentials (Omedes et al., 2013). However, the Bayes factor analysis for the mean amplitude around ERN provides moderate evidence for the existence of differences between correct and error trials. Even so, there were neither evident ERN nor CRN peaks. Therefore, our data seem to be more suitable for frequency analysis, which is more robust in such circumstances.

We found a midfrontal theta power increase following erroneous responses, in agreement with previous studies (Cavanagh, Zambrano-Vazquez & Allen, 2012; Chavarriaga, Sobolewski & Millán, 2014; Cohen, 2011; Pavone et al., 2016; Völker et al., 2018; Zhang et al., 2015). This result supports its role in signaling the need for enhanced control and attention allocation (Van Noordt et al., 2017). Following this alarm signal, the lateral prefrontal cortex implements adaptive changes in cognitive control to avoid further errors (Cavanagh, Zambrano-Vazquez & Allen, 2012; Cohen & Cavanagh, 2011).

On the other hand, before response initiation, we found the inverse pattern of theta modulation. The decrease in theta power during the preparation of an erroneous response suggests that enhanced theta is required for executive function success during challenging conditions, namely response inhibition to no-go and anti-saccade stimuli (Nigbur, Ivanova & Stürmer, 2011; Van Noordt et al., 2017). This is in line with the link between theta activity and attentional control (Cavanagh, Zambrano-Vazquez & Allen, 2012; Van Noordt et al., 2017). When there is a demand for attention, the theta power appears to increase; and a decrease in theta activity before poor participant performance might occur due to attentional lapses (Van Noordt et al., 2017).

Despite sparse evidence, the theta band decrease preceding erroneous responses has already been suggested in other contexts (Atchley, Klee & Oken, 2017; Cavanagh, Cohen & Allen, 2009). Previously, this finding was shown before errors induced by boredom and mind-wandering (Atchley, Klee & Oken, 2017) and during a Flanker task (Cavanagh, Cohen & Allen, 2009). Van Noordt et al. (2017) also suggested reduced theta activity before errors in a saccade task, but only before anti-saccade trials. Similarly, event-related potential and fMRI studies have identified signatures of pre-error neural activity. Ray Li et al. (2007) have shown that errors are preceded by increased DMN activity; and a few studies have described the error-preceding positivity as an event-related potential that arises before erroneous actions (Hajcak et al., 2005; Hoonakker, Doignon-Camus & Bonnefond, 2016; Schroder et al., 2017). Pre-error brain activity has also been described in other studies (Bengson, Mangun & Mazaheri, 2012; Britz & Michel, 2010; Eichele et al., 2010; Maidhof et al., 2009; Pourtois, 2011; Ruiz, Jabusch & Altenmuller, 2009). Therefore, it would be important to explore the relationship between the theta band decreased activity preceding errors and both the error-preceding positivity and the DMN increased activity in future studies.

Taken together, the midfrontal theta patterns revealed by our results regarding response preparation and execution fit previous suggestions of theta oscillations as an indicator of the ability to establish appropriate response control. Increased theta activity has been related to the increased mental activity (Atchley, Klee & Oken, 2017). Moreover, performance monitoring, as manifested by midfrontal theta power, has been described as a neuronal marker of continuous vigilance processes modulated by errors (Cavanagh, Zambrano-Vazquez & Allen, 2012; Vlamings et al., 2008).

Since impairments in error monitoring processes are associated with several brain disorders (Bates et al., 2009; Olvet & Hajcak, 2008; Santesso et al., 2011), this neuronal marker might help to study and understand them. For instance, the impairments in error monitoring observed in ASD are believed to contribute to the observed repetitive behavior and social deficits (Santesso et al., 2011). Given that our task was specifically focused on the process of error monitoring during the integration of facial expression cues, and taking into account the ASD abnormal processing of faces (Pereira et al., 2019), it would be relevant to study the ASD error monitoring system using this task. Moreover, in the future, the theta band power could be used as a target for neurofeedback approaches aimed at improving response monitoring abilities.

Lastly, our study demonstrates that errors can be detected through midfrontal theta band power even when there is no clear ERN signature, which highlights the relevance of studying frequency patterns during complex scenarios. The great majority of studies concerning the neural correlates of error monitoring are based on controlled conditions using simple tasks and time-locked events, leading to high signal-to-noise ratios and avoiding typical confounds of realistic scenarios (Chavarriaga, Sobolewski & Millán, 2014). Our experiment was based on a go/no-go saccade task and, given that saccades are semi-automatic oculomotor responses to visual stimuli, there was a high variability of response timing. This context, with asynchronous saccades and facial cues, is hence more realistic than the usual simple tasks. Given that errors without a clear ERN were distinguished through theta band activity, our results support the notion that oscillatory activity allows asynchronous recognition of erroneous actions (Chavarriaga, Sobolewski & Millán, 2014; Spüler & Niethammer, 2015). This finding provides an important contribution to some neuroengineering systems, as is the case of brain-computer interfaces (BCI) (Chavarriaga, Sobolewski & Millán, 2014). Event-related potentials related to error monitoring have been applied in BCI output optimization (Plewan et al., 2016). In the future, error detection might be improved through theta power analysis. Moreover, the theta power modulation preceding responses might be used as a feature to predict errors, allowing faster and more efficient algorithms.

## Conclusions

We investigated the neuronal basis of error monitoring, before and after response execution, at the level of theta band activity. We aimed to contribute to the understanding of the role of theta power in self-monitoring of task performance during a saccade go/no-go task triggered by the integration of facial cues. We followed the hypothesis that this process could potentially modulate the theta power before and after a given response. Accordingly, midfrontal theta power was found to signal not only the reaction to error events but also possible failures of attentional focus during the preparation time before responses. We found a decreased mid-frontal theta activity before erroneous responses and an increase in post-error theta power. These findings help to clarify the contribution of the theta band activity to error monitoring and highlight the relevance of studying the modulation of frequency patterns during such a process.

Given that the major limitation of this study is the number of errors, the paradigm could be optimized in terms of task difficulty to elicit a higher number of errors and to control for non-saccadic responses. With more errors, some differences between error types—go pro and anti, and no-go trials—might also potentially be discovered and the relation between theta and error-related potentials further explored. Moreover, a task requiring other response modalities will contribute to clarify the pre- and post-response midfrontal theta band modulation during performance monitoring.

## Supplemental Information

10.7717/peerj.12627/supp-1 Supplemental Information 1 Saccadic movement detection The diagram depicts the saccade detection algorithm used. It illustrates saccades (green) - (A) and (C) - and small ocular/head movements, micro-saccades or blinks (red) - (B), (D) and (E). (A) and (C) are considered saccades since, on the one hand, they have a horizontal ocular movement amplitude superior to 4.04 of visual angle and, on the other, they do not finish their trajectory in the central area (which is characterized by a distance of 4.04 of visual angle from the center). The movement illustrated on (B), on the contrary, finishes its path in the central area. Otherwise, in (D) it does not, but its amplitude is inferior to 4.04. Lastly, (E) possibly represents a blink, because its vertical amplitude is higher than its horizontal length.

10.7717/peerj.12627/supp-2 Supplemental Information 2 Average FCz amplitude for correct and error trials Our main research question was related to frequency analysis, and our paradigm was optimized to study the theta band activity. However, we also investigated the EEG signal in the time domain, and here the FCz amplitude around the ERN position.

10.7717/peerj.12627/supp-3 Supplemental Information 3 Average Pz amplitude for correct and error trials Our main research question was related to frequency analysis, and our paradigm was optimized to study the theta band activity. However, we also investigated the EEG signal in the time domain, and here the Pz amplitude around the Pe position.

10.7717/peerj.12627/supp-4 Supplemental Information 4 Statistical results of the behavioral analysis (comparison between correct and erroneous responses) For each metric (response timing, saccade amplitude, and saccade duration), 20 tests were performed to balance correct and error trials. For each test, the test statistic, *p*-value (significant values are represented in bold) and effect size are presented. To consider a difference as statistically significant, 80% of the tests must be characterized by *p* ≤ 0.05.

10.7717/peerj.12627/supp-5 Supplemental Information 5 Statistical results of the neurophysiological analysis (comparison between correct and erroneous responses) For each metric (ERN amplitude, Pe amplitude, theta-band activity before response and theta-band activity after response), 20 tests were performed to balance correct and error trials. For each test, the test statistic, *p*-value (significant values are represented in bold) and effect size are presented. To consider a difference as statistically significant, 80% of the tests must be characterized by *p* ≤ 0.05.

## Additional Information and Declarations

### Data Availability

The following information was supplied regarding data availability: The data are available at Zenodo: Dias, Camila, Costa, Diana M., Sousa, Teresa, Castelhano, João, Figueiredo, Verónica, Pereira, Andreia C., & Castelo-Branco, Miguel. (2021). EEG and eye-tracking data from a go/no-go saccadic task based on facial expression cues [Data set]. Zenodo. https://doi.org/10.5281/zenodo.5608640.
