# Supplementary figures and images for: A neuronal theta band signature of error monitoring during integration of facial expression cues
Source: PeerJ. 2022 Feb 17;10:e12627. doi: 10.7717/peerj.12627 (PMC8858578; doi:10.7717/peerj.12627)

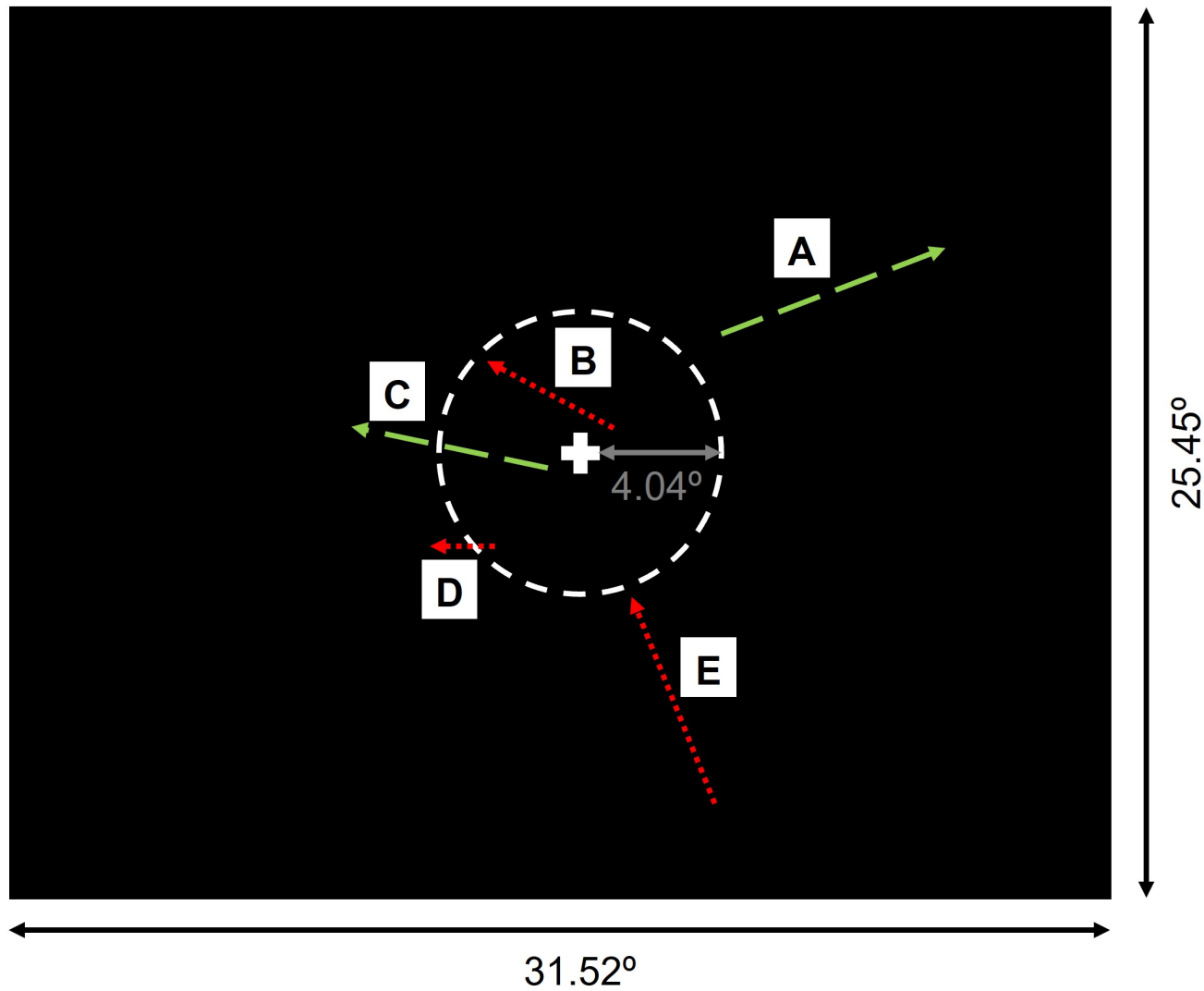

Supplement: Supplemental Information 1 — The diagram depicts the saccade detection algorithm used. It illustrates saccades (green) - (A) and (C) - and small ocular/head movements, micro-saccades or blinks (red) - (B), (D) and (E). (A) and (C) are considered saccades since, on the one hand, they have a horizontal ocular movement amplitude superior to 4.04 of visual angle and, on the other, they do not finish their trajectory in the central area (which is characterized by a distance of 4.04 of visual angle from the center). The movement illustrated on (B), on the contrary, finishes its path in the central area. Otherwise, in (D) it does not, but its amplitude is inferior to 4.04. Lastly, (E) possibly represents a blink, because its vertical amplitude is higher than its horizontal length. [file peerj-10-12627-s001.pdf]

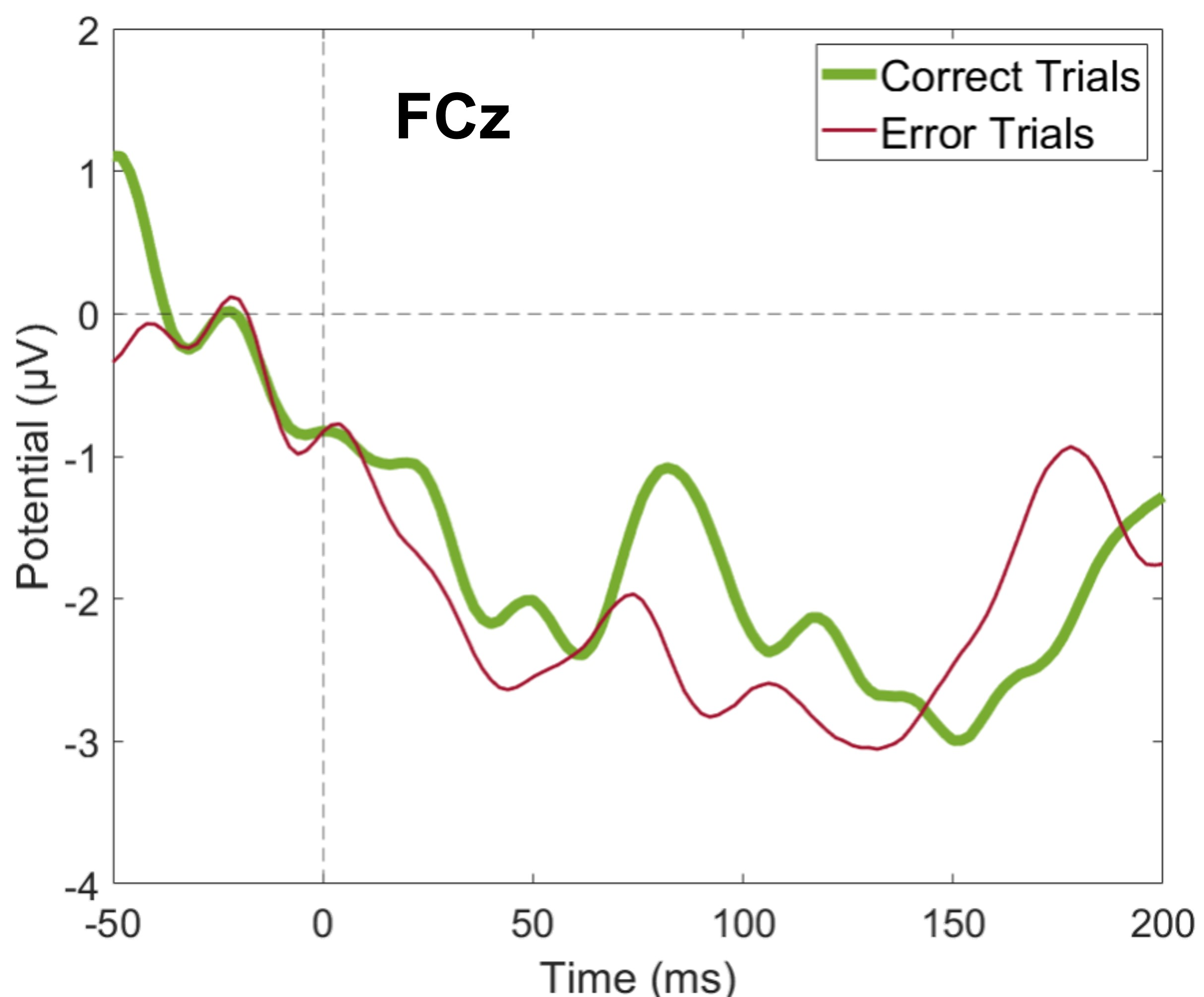

Supplement: Supplemental Information 2 — Our main research question was related to frequency analysis, and our paradigm was optimized to study the theta band activity. However, we also investigated the EEG signal in the time domain, and here the FCz amplitude around the ERN position. [file peerj-10-12627-s002.png]

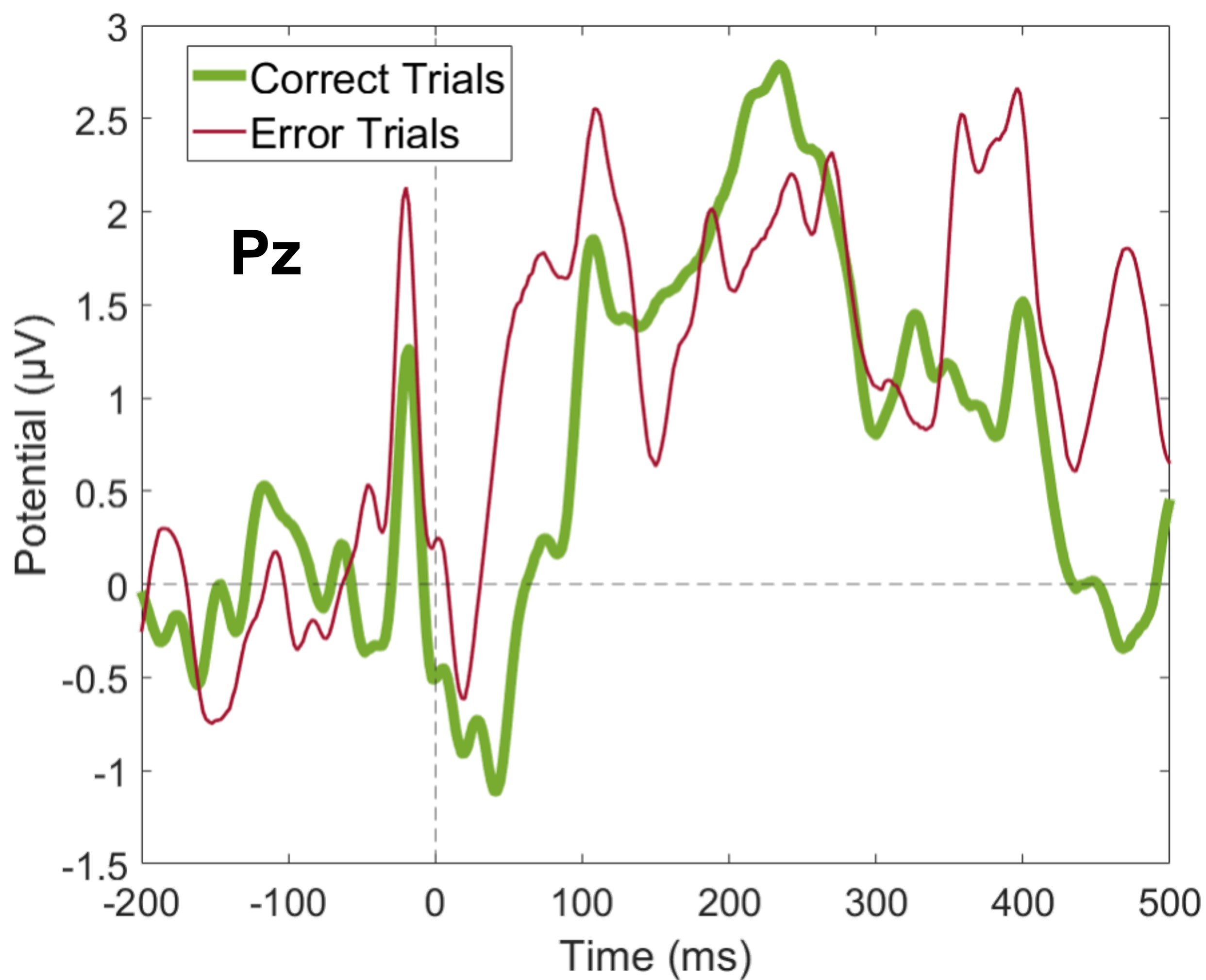

Supplement: Supplemental Information 3 — Our main research question was related to frequency analysis, and our paradigm was optimized to study the theta band activity. However, we also investigated the EEG signal in the time domain, and here the Pz amplitude around the Pe position. [file peerj-10-12627-s003.png]
