# Supplementary material for: A neuronal theta band signature of error monitoring during integration of facial expression cues
Source: PeerJ. 2022 Feb 17;10:e12627. doi: 10.7717/peerj.12627 (PMC8858578; doi:10.7717/peerj.12627)
Supplement: Supplemental Information 4 — For each metric (response timing, saccade amplitude, and saccade duration), 20 tests were performed to balance correct and error trials. For each test, the test statistic, p-value (significant values are represented in bold) and effect size are presented. To consider a difference as statistically significant, 80% of the tests must be characterized by p ≤ 0.05. [file peerj-10-12627-s004.docx]

|  | **Response timing** | | | **Saccade amplitude** | | | **Saccade duration** | | |
| --- | --- | --- | --- | --- | --- | --- | --- | --- | --- |
| Test | ***Z*** | ***p*** | ***r*** | ***Z*** | ***p*** | ***r*** | ***Z*** | ***p*** | ***r*** |
| 1 | -0.68 | 0.49 | -0.16 | -2.09 | **0.04 (*)** | -0.48 | -2.82 | **0.01 (*)** | -0.65 |
| 2 | -0.52 | 0.60 | -0.12 | -2.05 | **0.04 (*)** | -0.47 | -2.74 | **0.01 (*)** | -0.63 |
| 3 | -0.64 | 0.52 | -0.15 | -2.29 | **0.02 (*)** | -0.53 | -2.29 | **0.02 (*)** | -0.53 |
| 4 | -0.52 | 0.60 | -0.12 | -2.09 | **0.04 (*)** | -0.48 | -2.05 | **0.04 (*)** | -0.47 |
| 5 | -1.17 | 0.24 | -0.27 | -1.77 | 0.08 | -0.41 | -2.54 | **0.01 (*)** | -0.58 |
| 6 | -0.64 | 0.52 | -0.15 | -2.82 | **0.01 (*)** | -0.65 | -2.21 | **0.03 (*)** | -0.51 |
| 7 | -1.89 | 0.06 | -0.43 | -1.81 | 0.07 | -0.42 | -3.30 | **0.001 (*)** | -0.76 |
| 8 | -1.13 | 0.26 | -0.26 | -1.89 | 0.06 | -0.43 | -2.37 | **0.02 (*)** | -0.54 |
| 9 | -1.21 | 0.23 | -0.28 | -2.66 | **0.01 (*)** | -0.61 | -1.89 | 0.06 | -0.43 |
| 10 | -0.48 | 0.63 | -0.11 | -2.37 | **0.02 (*)** | -0.54 | -1.57 | 0.12 | -0.36 |
| 11 | -0.77 | 0.45 | -0.18 | -2.37 | **0.02 (*)** | -0.54 | -2.21 | **0.03 (*)** | -0.51 |
| 12 | -0.56 | 0.57 | -0.13 | -1.69 | 0.09 | -0.39 | -2.55 | **0.01 (*)** | -0.58 |
| 13 | -1.09 | 0.28 | -0.25 | -2.29 | **0.02 (*)** | -0.53 | -2.29 | **0.02 (*)** | -0.53 |
| 14 | -1.01 | 0.31 | -0.23 | -1.53 | 0.13 | -0.35 | -2.25 | **0.02 (*)** | -0.52 |
| 15 | -0.68 | 0.49 | -0.16 | -1.85 | 0.06 | -0.42 | -2.50 | **0.01 (*)** | -0.57 |
| 16 | -0.97 | 0.33 | -0.22 | -1.61 | 0.11 | -0.37 | -1.73 | 0.08 | -0.40 |
| 17 | -0.48 | 0.63 | -0.11 | -2.09 | **0.04 (*)** | -0.48 | -2.29 | **0.02 (*)** | -0.53 |
| 18 | -1.69 | 0.09 | -0.39 | -1.45 | 0.15 | -0.33 | -2.54 | **0.01 (*)** | -0.58 |
| 19 | -0.85 | 0.40 | -0.19 | -1.61 | 0.11 | -0.37 | -2.13 | **0.03 (*)** | -0.49 |
| 20 | -0.64 | 0.52 | -0.15 | -2.25 | **0.02 (*)** | -0.52 | -2.62 | **0.01 (*)** | -0.60 |

Table S1: **Statistical results of the behavioral analysis (comparison between correct and erroneous responses).** For each metric (response timing, saccade amplitude and saccade duration), 20 tests were performed to balance correct and error trials. For each test, the test statistic, *p*-value (significant values are represented in bold) and effect size are presented. To consider a difference as statistically significant, 80% of the tests must be characterized by *p* ≤ 0.05.
