# Supplementary material for: A neuronal theta band signature of error monitoring during integration of facial expression cues
Source: PeerJ. 2022 Feb 17;10:e12627. doi: 10.7717/peerj.12627 (PMC8858578; doi:10.7717/peerj.12627)
Supplement: Supplemental Information 5 — For each metric (ERN amplitude, Pe amplitude, theta-band activity before response and theta-band activity after response), 20 tests were performed to balance correct and error trials. For each test, the test statistic, p-value (significant values are represented in bold) and effect size are presented. To consider a difference as statistically significant, 80% of the tests must be characterized by p ≤ 0.05. [file peerj-10-12627-s005.docx]

|  | **ERN** | | | **Pe** | | | **Theta before** | | | **Theta after** | | |
| --- | --- | --- | --- | --- | --- | --- | --- | --- | --- | --- | --- | --- |
| Test | ***Z*** | ***p*** | ***r*** | ***Z*** | ***p*** | ***r*** | ***Z*** | ***p*** | ***r*** | ***Z*** | ***p*** | ***r*** |
| 1 | -1.53 | 0.13 | -0.35 | -1,89 | 0,06 | -0,43 | -2.84 | **0.01 (*)** | -0.65 | -1.90 | 0.06 | -0.44 |
| 2 | -1.45 | 0.15 | -0.33 | -1,29 | 0,20 | -0,30 | -2.13 | **0.03 (*)** | -0.49 | -1.98 | **0.05 (*)** | -0.45 |
| 3 | -1.21 | 0.23 | -0.28 | -1,97 | **0,05 (*)** | -0,45 | -1.87 | 0.06 | -0.43 | -2.24 | **0.03 (*)** | -0.51 |
| 4 | -0.81 | 0.42 | -0.18 | -2,25 | **0,02 (*)** | -0,52 | -2.73 | **0.01 (*)** | -0.63 | -2.13 | **0.03 (*)** | -0.49 |
| 5 | -1.45 | 0.15 | -0.33 | -2,33 | **0,02 (*)** | -0,54 | -1.57 | 0.12 | -0.36 | -2.69 | **0.01 (*)** | -0.62 |
| 6 | -1.77 | 0.08 | -0.41 | -2,01 | **0,04 (*)** | -0,46 | -1.98 | **0.05 (*)** | -0.45 | -2.73 | **0.01 (*)** | -0.63 |
| 7 | -1.81 | 0.07 | -0.42 | -2,58 | **0,01 (*)** | -0,59 | -2.35 | **0.02 (*)** | -0.54 | -2.61 | **0.01 (*)** | -0.60 |
| 8 | -0.36 | 0.72 | -0.08 | -1,73 | 0,08 | -0,40 | -1.16 | 0.25 | -0.27 | -2.24 | **0.03 (*)** | -0.51 |
| 9 | -1.05 | 0.30 | -0.24 | -2,29 | **0,02 (*)** | -0,53 | -2.17 | **0.03 (*)** | -0.50 | -2.28 | **0.02 (*)** | -0.52 |
| 10 | -0.52 | 0.60 | -0.12 | -2,78 | **0,01 (*)** | -0,64 | -2.65 | **0.01 (*)** | -0.61 | -2.43 | **0.02 (*)** | -0.56 |
| 11 | -1.69 | 0.09 | -0.39 | -2,66 | **0,01 (*)** | -0,61 | -2.17 | **0.03 (*)** | -0.50 | -2.05 | **0.04 (*)** | -0.47 |
| 12 | -1.01 | 0.31 | -0.23 | -1,37 | 0,17 | -0,31 | -2.80 | **0.01 (*)** | -0.64 | 2.35 | **0.02 (*)** | 0.54 |
| 13 | -1.29 | 0.20 | -0.30 | -2,17 | **0,03 (*)** | -0,50 | -2.91 | **0.004 (*)** | -0.67 | -2.39 | **0.02 (*)** | -0.55 |
| 14 | -1.93 | 0.05 | -0.44 | -1,53 | 0,13 | -0,35 | -2.65 | **0.01 (*)** | -0.61 | -2.13 | **0.03 (*)** | -0.49 |
| 15 | -0.40 | 0.69 | -0.09 | -2,58 | **0,01 (*)** | -0,59 | -2.39 | **0.02 (*)** | -0.55 | -2.80 | **0.01 (*)** | -0.64 |
| 16 | -0.36 | 0.72 | -0.08 | -2,46 | **0,01 (*)** | -0,56 | -2.69 | **0.01 (*)** | -0.62 | -2.09 | **0.04 (*)** | -0.48 |
| 17 | -1.17 | 0.24 | -0.27 | -1,89 | 0,06 | -0,43 | -2.84 | **0.01 (*)** | -0.65 | -2.65 | **0.01 (*)** | -0.61 |
| 18 | -1.41 | 0.16 | -0.32 | -2,37 | **0,02 (*)** | -0,54 | -2.20 | **0.03 (*)** | -0.51 | -1.79 | 0.07 | -0.41 |
| 19 | -1.37 | 0.17 | -0.31 | -1,57 | 0,12 | -0,36 | -2.13 | **0.03 (*)** | -0.49 | -2.84 | **0.01 (*)** | -0.65 |
| 20 | -0.28 | 0.78 | -0.06 | -2,78 | **0,01 (*)** | -0,64 | -1.72 | 0.09 | -0.39 | -2.50 | **0.01 (*)** | -0.57 |

Table S2: **Statistical results of the neurophysiological analysis (comparison between correct and erroneous responses).** For each metric (ERN amplitude, Pe amplitude, theta-band activity before response and theta-band activity after response), 20 tests were performed to balance correct and error trials. For each test, the test statistic, *p*-value (significant values are represented in bold) and effect size are presented. To consider a difference as statistically significant, 80% of the tests must be characterized by *p* ≤ 0.05.
